# Lasers are superfluous for the surgical management of benign prostatic hyperplasia in the developing world

**DOI:** 10.4103/0970-1591.56190

**Published:** 2009

**Authors:** Narmada P. Gupta, Ajay Anand

**Affiliations:** Department of Urology, All India Institute of Medical Sciences, New Delhi 110 029, India

**Keywords:** BPH, lasers

## Abstract

Lasers have been given much hype as regards their use in surgical management of benign prostatic hyperplasia (BPH). Transurethral resection of prostate (TURP), especially with its modifications still remains the gold standard treatment for BPH, owing to its efficacy and proven advantages over laser prostatectomy. Cost, unproven long-term durability, steep learning curve, and no advantages of laser prostatectomy over TURP and its modifications, make lasers superfluous in the surgical management of BPH in developing countries.

## INTRODUCTION

Lasers have been given much hype with regard to their use in the surgical management of BPH (Benign Prostatic hyperplasia). It seems to be too early to comment on their effectiveness in view of the established fact that TURP(Transurethral Resection of prostate) is considered the gold standard treatment against which all other surgical modalities of treatment are weighed. TURP has stood the test of time and is the most commonly performed surgical procedure for BPH all over the world. TURP was criticized because of its complications as reported by Mebust in 1989.[1] The common complications are hemorrhage, fluid absorption, and TURP syndrome. There has been a search for the replacement of TURP by various minimally invasive procedures including lasers but none have proven their superiority over TURP so far.

There have been modifications in TURP such as the use of thick loops and the use of bipolar energy to reduce its complications, shorter catheter duration, and hospital stay. TUVRP (Transurethral Vapor resection of Prostate), which uses thick loop wing cut electrodes, is an advancement over TURP.[2] TUVRP has significant advantages over TURP including better tissue removal, less bleeding, greater visibility, better hemostasis, shorter operative time, shorter duration of postoperative irrigation and catheterization, and shorter hospital stay. TUVRP leads to simultaneous resection as well as vaporization, with about 50% of estimated resected weight of prostate being retrieved as chips.[3] Lasers require a longer time for resection/enucleation of prostates and some lasers, such as KTP (Potassium Titanyl Phosphate) are not considered good as there is only vaporization of the tissue and it may not be effective for larger prostates. This becomes all the more important in view of our observation that most patients are presenting for surgical treatment with large size prostates, usually more than 50 grams, unlike in the past when prostates presented for surgical management were usually less than 50 grams. Holmium laser enucleation of the prostate (HOLEP) has been considered to be effective in larger prostates, yet it is not cost-effective and the resection time is longer than TUVRP. TUVRP requires less time for surgery than standard TURP.[3]

We compared standard TURP, TUVRP, and holmium laser resection/enucleation of the prostate. This study replicated the results of the previous study and revealed the excellent hemostatic property of the holmium laser with less perioperative morbidity. However, the laser surgery requires special equipment and is associated with a learning curve.[4]

We also studied the role of TUVRP in prostates weighing more than 70 grams and found that any size prostate can be easily resected with minimal morbidity. TUVRP provides fast resectability of the prostate adenoma with good homeostasis and a short hospital stay.[5]

Various groups have reported other alternatives such as HOLEP for surgical management of a large volume prostate.[6–11] HOLEP requires surgical expertise and special equipment, which is available only in a few centers worldwide. With the use of bipolar energy and saline for irrigation, the size of the prostate gland is no longer a limitation for TURP. Other advantages of bipolar TURP are less bleeding, short catheter time, and shorter hospital stay. Bipolar TURP is a safe and effective procedure associated with fewer side effects and could result in the procedure being more attractive for high-risk patients, as well as for training purposes.[12]

The cost and availability of the equipment is an important factor in developing countries. The initial cost of laser machines is high and the cost of fiber is added in every case. In the case of laser enucleation, bare fibers can be reused but in vaporization procedures, one or sometimes more than one fiber is required. Apart from this, there is a high maintenance cost and additional hazards associated with laser machines. In comparison, TURP equipment is easily available, cheap with minimum maintenance cost, and is available with every urologist as basic equipment.

It is important that a surgical procedure should be durable and not require repeat procedures for the same disease. The long-term outcomes of TURP are good. Patients who undergo laser vaporization may require repeat procedure/TURP during follow-up. Vavassori reported a re-intervention rate of 2.7% for residual adenoma whereas Elzayat and Elhilali reported a re-treatment rate of 8% in their series.[11,13] The large variety of lasers and techniques of laser prostatectomy shows that none is perfect so far. There is a steep learning curve for laser prostatectomy and this is one of the reasons why its use is limited to a few hands and a few centers.

## CONCLUSION

Lasers are superfluous in the surgical management of BPH in developing countries because of the cost, unproven long-term durability, steep learning curve, and no advantages over the gold standard management of TURP, especially with its modifications.
